# Comparison of a Clinical Prediction Rule and a LAM Antigen-Detection Assay for the Rapid Diagnosis of TBM in a High HIV Prevalence Setting

**DOI:** 10.1371/journal.pone.0015664

**Published:** 2010-12-22

**Authors:** Vinod B. Patel, Ravesh Singh, Cathy Connolly, Victoria Kasprowicz, Allimudin Zumla, Thumbi Ndungu, Keertan Dheda

**Affiliations:** 1 Department of Neurology, University of KwaZulu Natal, Berea, South Africa; 2 HIV Pathogenesis Programme, Doris Duke Medical Research Institute, Nelson R. Mandela School of Medicine, University of KwaZulu Natal, Berea, South Africa; 3 Biostatistics Unit, Medical Research Council, Durban, South Africa; 4 Department of Infection, Centre for Infectious Diseases and International Health, University College London, London, United Kingdom; 5 Lung Infection and Immunity Unit, Division of Pulmonology and Department of Medicine, University of Cape Town Lung Institute, University of Cape Town, Cape Town, South Africa; 6 Institute of Infectious Disease and Molecular Medicine, University of Cape Town, Cape Town, South Africa; University of Stellenbosch, South Africa

## Abstract

**Background/Objective:**

The diagnosis of tuberculous meningitis (TBM) in resource poor TB endemic environments is challenging. The accuracy of current tools for the rapid diagnosis of TBM is suboptimal. We sought to develop a clinical-prediction rule for the diagnosis of TBM in a high HIV prevalence setting, and to compare performance outcomes to conventional diagnostic modalities and a novel lipoarabinomannan (LAM) antigen detection test (Clearview-TB®) using cerebrospinal fluid (CSF).

**Methods:**

Patients with suspected TBM were classified as definite-TBM (CSF culture or PCR positive), probable-TBM and non-TBM.

**Results:**

Of the 150 patients, 84% were HIV-infected (median [IQR] CD4 count = 132 [54; 241] cells/µl). There were 39, 55 and 54 patients in the definite, probable and non-TBM groups, respectively. The LAM sensitivity and specificity (95%CI) was 31% (17;48) and 94% (85;99), respectively (cut-point ≥0.18). By contrast, smear-microscopy was 100% specific but detected none of the definite-TBM cases. LAM positivity was associated with HIV co-infection and low CD4 T cell count (CD4<200 vs. >200 cells/µl; p = 0.03). The sensitivity and specificity in those with a CD4<100 cells/µl was 50% (27;73) and 95% (74;99), respectively. A clinical-prediction rule ≥6 derived from multivariate analysis had a sensitivity and specificity (95%CI) of 47% (31;64) and 98% (90;100), respectively. When LAM was combined with the clinical-prediction-rule, the sensitivity increased significantly (p<0.001) to 63% (47;68) and specificity remained high at 93% (82;98).

**Conclusions:**

Despite its modest sensitivity the LAM ELISA is an accurate rapid rule-in test for TBM that has incremental value over smear-microscopy. The rule-in value of LAM can be further increased by combination with a clinical-prediction rule, thus enhancing the rapid diagnosis of TBM in HIV-infected persons with advanced immunosuppression.

## Introduction

Although the tuberculosis (TB) epidemic has plateaued in several regions of the world, in Africa, fuelled by poverty and HIV co-infection, TB is out of control. South Africa has the fifth highest burden of TB and the largest number of HIV-infected residents in any one country worldwide [1]. Given the high rate of HIV-TB co-infection, extra-pulmonary TB (EPTB) and hence central nervous system TB, which comprises 1 to 18% of EPTB [2], [3], [4], [5], is a common clinical problem.

HIV-infected patients with TB meningitis (TBM) are particularly challenging to manage because there are no accurate tools to rapidly establish a diagnosis and delay in establishing treatment is associated with mortality [6], [7], [8]. Smear-microscopy in an ideal research setting, and using high volumes of processed CSF, may have a modest sensitivity [8], [9]. However, in a programmatic setting in Africa the yield is dismal and recent studies in HIV-infected populations reveal a sensitivity of less than 5% [10]. Polymerase chain reaction (PCR), which may be used as a confirmatory test for TBM [11], is a good rapid rule-in test for TBM with a sensitivity of ∼40 to 50% but this technology is unavailable in most hospitals in Africa [12]. We recently showed that CSF antigen-specific quantitative T cell assays may be a rapid and accurate rule-in test for TBM but the available ELISPOT assay (T SPOT TB) is expensive and requires overnight processing [13]. The initial promise of antibody [8], [14], [15] and antigen-based [16], [17], [18] tests for TBM have not been sustained. Given the poor performance of diagnostic tools we attempted to devise a clinical prediction rule, hitherto unavailable, suited to resource-poor high HIV prevalence settings.

Very recently a standardised lipoarabinomannan (LAM) antigen-detection ELISA test, which yields a result within 2 to 3 hours, has been developed (Clearview® TB *ELISA*, ME, USA; see http://www.clearview.com/tb_elisa.aspx) and is useful for the diagnosis of TB in HIV-infected persons with advanced immunosuppression [19], [20], [21]. Although tested mainly in urine [22], [23], [24], [25], [26], we recently evaluated this assay in a proof-of-concept study using CSF from patients with suspected TBM [27]. Given the promising results in CSF [27] we performed a prospective study using the LAM Clearview TB® ELISA test in a predominantly HIV-infected population. To our knowledge there are no previous reports evaluating this assay in CSF. A particularly interesting feature of this technology, which highlights the broader significance of this work, is that a point-of-care (POC) lateral flow assay has been developed and is currently being evaluated in clinical trials [21].

## Methods

### Patient recruitment and processing

One hundred and fifty consecutive patients were prospectively recruited over a period of 15 months, between January 2008 and April 2009, at Inkosi Albert Luthuli Central Hospital (IALCH), a tertiary referral center in Durban, South Africa. This study was approved by the biomedical research ethics committee of the University of KwaZulu-Natal. Patients presenting with a meningeal illness indicating the need for a lumbar puncture (LP) were recruited from referring regional hospitals. Detailed recruitment and patient processing methods were recently described [28]. Informed written consent was obtained from all patients (in patients who were unable provide consent at initial presentation, due to an abnormal mental state, consent was obtained from a first degree relative or from the Head of Department when a lumbar puncture was clinically justified) [29]. After excluding contraindications to a lumbar puncture (LP), CSF samples were processed for microscopy (auramine staining of centrifuged samples using a mercury vapour fluorescent microscope), *Mycobacterium tuberculosis* (*M.tb*), bacterial, and fungal culture, and tests were performed to exclude other locally prevalent causes of meningitis including microscopy (Gram stain and for acid-fast bacilli), routine chemistry (protein, glucose, chloride), TB PCR (Roche Amplicor, Roche Diagnostics GmbH, Roche Applied Science, 68298 Mannheim, Germany), viral PCR (Roche Amplicor) for [cytomegalovirus (CMV), herpes simplex (HSV type 1) and varicella zoster virus (VZV)], fluorescent treponemal antibody (FTA) test and venereal disease research laboratory (VDRL) for neurosyphilis if FTA was positive, cysticercal enzyme linked immunosorbent assay (ELISA), and a cryptococcal antigen latex agglutination test (CLAT) which has a high specificity and sensitivity. Detailed methods were described in a previous publication [28]. Blood for CD4 counts was taken and HIV status noted in all patients. Clinical information recorded included demographic information, duration of symptoms and anti-tuberculous therapy, HIV status, duration of steroid therapy, past history of tuberculosis and history of tuberculosis (TB) contact.

### Categorisation of patients

Patients were categorised as definite TBM (either CSF culture or PCR positive for *M.tb*) [11], probable TBM (clinical features of meningitis, an LP consistent with an aseptic bacterial meningitis, negative for other causes of meningitis, and two of the following: a chest X-ray consistent with active PTB, a CT scan consistent with TBM (basal enhancement or hydrocephalus), and a response to anti-tuberculous therapy), or non TBM (an alternate definite cause for meningitis identified and response to appropriate non-tuberculous therapy) [11], [30]. The reference standard was thus PCR or culture positivity for *M. tb* [11].

The laboratory technician was blinded to the clinical diagnosis and clinician blinded to the laboratory result. Tests were done in duplicate. Standard curves were derived by serial dilution of LAM antigen using CSF from a patient with benign intracranial hypertension.

### Laboratory processing

CSF samples were processed for the detection of *M.tb* using a standardized PCR assay (Roche AMPLICOR) as per manufacturer instructions. LAM antigen was measured using an ELISA kit (Clearview® TB ELISA, Inverness Medical Innovations, USA). The samples were thawed and allowed to equilibrate to room temperature. After an initial heating step (95–100°C for 30 min) to separate antigen-antibody complexes, CSF samples (0.2 mls) were seeded, in duplicate, into 96 well plates coated with anti-LAM antibodies. Following this an ELISA was done to measure optical density (OD) determined by a trained technician blinded to patient details. The LAM OD values were extrapolated from a standard curve constructed from two-fold serial dilutions (8 in total ranging from 10 to 0.08 mg/ml) of the LAM antigen (20 mg/ml) in CSF (Inverness Medical Innovations, USA).

### Statistical Analysis

Chi square tests or Fisher exact tests were used to compare categorical variables between TBM and non TBM patients. Numeric variables were compared using a t-test or Wilcoxin Rank sum test/or Kruskal Wallis test if normality could not be assumed. Diagnostic performance, including 95% confidence intervals was assessed using sensitivity, specificity, agreement (proportion in whom both sample-specific results were concordant), predictive values and area under the receiver operating characteristic (ROC) curve where the combined results of culture or PCR was used as the gold standard to classify patients as definite TBM or non TBM. Three cut-off points were used: the laboratory standard, Youden index [31] and maximum specificity.

A clinical index was generated using a stepwise logistic regression model. Continuous variables such as clinical and laboratory parameters were dichotomised using ROC curves to identify cut-off points which maximised specificity prior to inclusion in the model. Rounded β-coefficients from the reduced model of significant variables were used to create a weighted clinical index. The index was then dichotomised and the sensitivity and specificity calculated. The sensitivity and specificity were recalculated using the revised clinical plus LAM index. A one sample *z statistic* was used to determine if adding LAM to the clinical index resulted in a significant change in diagnostic performance.

## Results

### Patient characteristics

**Figure 1:**
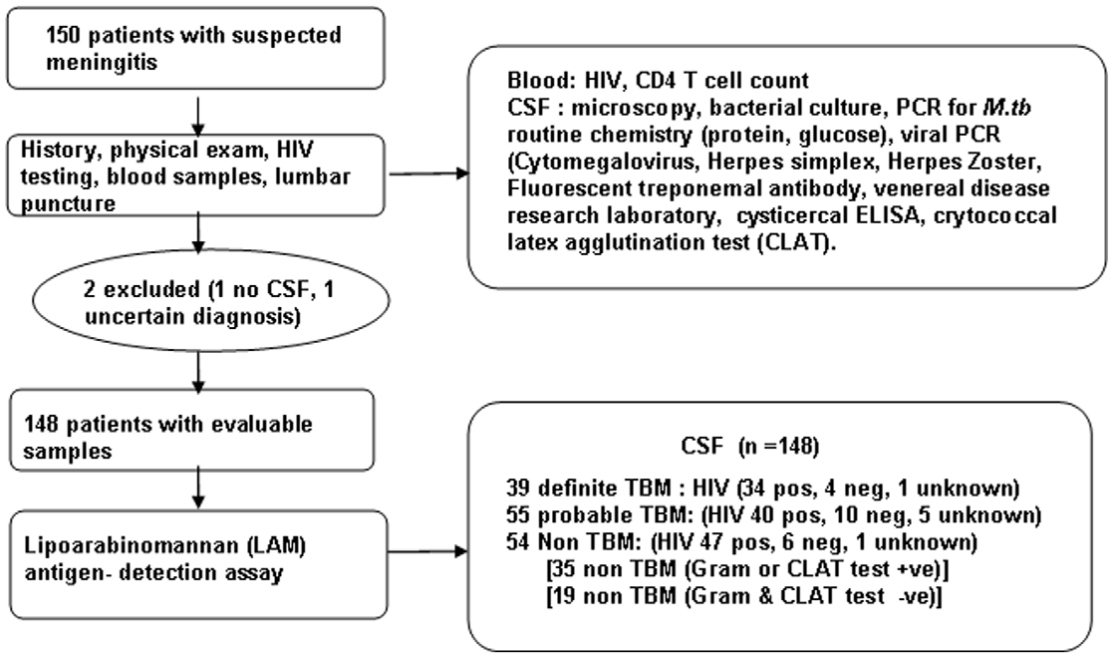
Summary flow chart of patient categorisation and investigations performed at recruitment.

Of the 150 consecutively recruited patients, two patients were excluded (in 1 patient no definite diagnosis made and in one other no CSF obtained). Thus, 148 samples were processed for LAM antigen (39 definite TBM [31 culture positive, 8 additional PCR positive patients), 55 probable TBM and 54 non TBM; Figure 1. There were 14 PCR positive samples within the cohort of 31 culture positive patients. 84% of the cohort was HIV-infected, 13.5% HIV uninfected, 5% declined HIV testing. The median inter-quartile range (IQR) CD4 count was 132 cells/µl (54;241). Socio-demographic and a comparison of CSF findings between definite TBM and non-TBM groups findings are shown in Table 1. Alternate diagnoses in the non TBM group included cryptococcal meningitis (n = 30), bacterial meningitis (n = 5), viral meningitis (n = 14; 4 VZV, 1 CMV, 9 unknown), neoplastic meningitis (n = 2), mucormycosis (n = 1), venous sinus thrombosis with CSF change (n = 1), and neurosyphilis (n = 1)

**Table 1:** Comparison of the clinical and laboratory parameters in the definite TB meningitis (culture or PCR positive; n = 39) and non TB meningitis (n = 54) groups.

Characteristic	Definite TBM (%) [IQR]	Non TBM (%) [IQR]	P value
	n (%)	n (%)	
Number	39 (42%)	54 (36%)	0.7
Mean age (±S.D)	33.5 (9.5)	32.9 (9.7)	0.7
Age			
<36/≥36 years*	24/15 (61.5/38.5)	35/19 (29.6/70.4)	0.7
Sex			
Male/Female	18/21 (46.2/53.9)	16/38 (29.6/70.4)	0.1
Ethnic Group			
BA/M/E/I	38/1/0/0 (97.4/2.6/0/0)	53/0/0/1 (98.2/0/0/1.9)	0.3
HIV status			
P/N/Unknown	34/4/1 (87.2/10.3/2.6)	47/6/1(87.0/11.1/1.9)	
Previous TB			
Yes/No/Unknown	8/27/4 (20.5/69.2/10.3)	24/30/0 (44.4/55.6/0)	0.007
TB contact (within 2 years)			
Yes/No/Unknown	9/26/4 (23.1/66.7/10.3	14/40/0 (25.9/74.1/0)	0.06
Duration of illness (days)			
<6/≥6 days*	6/31 (16.2/83.8)	9/45 (16.7/83.3)	0.9
Steroid treatment			
Yes/No	12/27 (30.8/69.2)	8/46 (14.8/85.2)	0.07
CLAT			
Yes/no	4/35 (10.3/89.7)	27/27 (50/50)	<0.001
CD4 cells/µl [IQR]	84 [53–173]	161 [54–261]	0.04
Hydrocephalus (CT/MRI)			
Yes/no	17/13 (56.7/43.3)	10/13 (43.5/56.5)	0.3

CSF parameters	Definite TBM†	Non TBM†	P value
Lymphocytes (cells/µl)	99 [16]–[42]	28 [10–82]	0.01
Neutrophils (cells/µl)	67 [20–134]	9 [0–66]	0.002
Protein g/l	1.8 [1.14–2.7]	1.0 [0.8–1.9]	<0.001
CSF Glucose mmol/l	1 [1.0–1.7]	2.1 [1.5–2.7]	<0.001
CSF: serum Glucose ratio	0.2 [0.16–0.3]	0.4 [0.2–0.5]	<0.001
Lymphocytes: total ratio	0.6 (0.2–0.8)	0.8 (0.2–1.0)	0.2

*We chose a 36 year and 6 day cut off as this was a significant discriminator.

between acute septic and aseptic meningitis [9].

BA (Black African), M (mixed race), E (European), I (Indian).

P (positive), N (negative).

† =  Median and inter-quartile ranges.

### LAM antigen detection test outcomes

**Figure 2:**
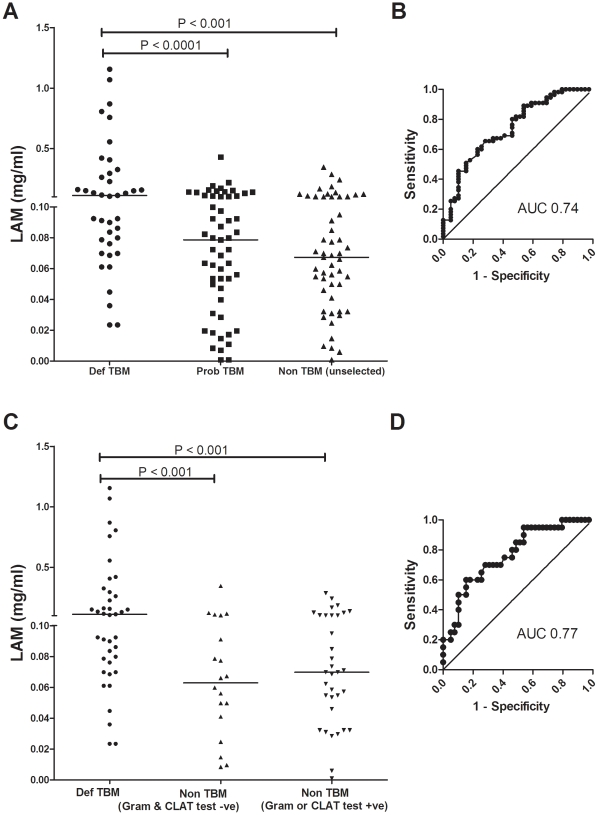
Lipoarabinomannan antigen performance outcomes using CSF when comparing definite, probable and non-TB meningitis groups. (A) shows the definite TBM compared with the unselected non-TB meningitis group and the corresponding ROC curve (B). Responses when the non-TB meningitis group was stratified by rapid test results (Gram stain or CLAT positive, versus, Gram stain and CLAT both negative) are shown in (C) with the corresponding ROC curve (D). Note (C) for the sake of clarity does not show the probable TB meningitis group.

Performance outcomes were derived using the manufacturer's recommended cut-point (OD value of 0.1295), an optimal cut-point using Youden's index (a point on the ROC curve yielding maximal sensitivity matched with the corresponding specificity) and the AUC-derived cut point selecting for high specificity at the expense of sensitivity. Outcomes are shown when the definite TBM group (n = 39) was compared to: (i) the entire non TBM group; i.e. including patients with cryptococcal, acute bacterial and other causes mentioned above (n = 54; Table 2A), (ii) non-TBM patients who were Gram stain or CLAT positive (n = 35; Table 2B), and (iii) non-TBM patients who were Gram stain or CLAT negative (n = 19; Table 2C). Thus, to evaluate whether other simultaneously used rapid tests impacted on test findings, we determined results for 3 different cut points in each of the categories specified. We selected a cut-point of ≥0.18 because a high specificity and moderate sensitivity defined a clinically useful rule-in test; Table 2. Figure 2 illustrates the comparison between definite and non TBM groups (unselected) and when the non TBM group was split into CLAT and Gram stain positive samples, and CLAT and Gram stain negative with the corresponding ROC curves.

**Table 2:** Performance outcomes (sensitivity, specificity, predictive values and accuracy) of the LAM ELISA (95% CI), at different cut-points in the definite TBM and non-TBM groups, using CSF.

2A. Definite TBM (n = 39) compared to unselected Non TBM (n = 54) AUC# = 0.74 (0.64;0.84)					
Cut Point (OD)	Sens (CI)	Spec (CI)	PPV (CI)	NPV (CI)	Agreement (CI)
≥0.1295*	69% (52;83)	65% (51;77)	59% (43;73)	74% (60;86)	67% (56;76)
≥0.148†	46% (30;63)	89% (77;96)	75% (53;90)	70% (57;80)	71% (61;80)
≥0.18‡	31% (17;48)	94% (85;99)	80% (52;96)	65% (54;76)	68% (57;77)

2B. Definite TBM (n = 39) compared to non TBM (n = 35) where non-TB samples were Gram stain or CLAT positive AUC 0.73 (0.61;0.84)					
Cut Point (OD)	Sens (CI)	Spec (CI)	PPV (CI)	NPV (CI)	Agreement (CI)
≥0.1295*	69% (52;83)	63% (45;79)	68% (51;81)	65%(46;80)	66% (54;77)
≥0.148†	46% (30;63)	86% (70;95)	78% (56;93)	59% (44;72)	65% (53;76)
≥0.18 ‡	31% (17;48)	94% (81;99)	86% (57;98)	55% (42;68)	61% (49;72)

2C. Definite TBM (n = 39) compared to non-TBM (n = 19) where non-TB samples were not Gram stain or CLAT positive AUC 0.77 = (0.63;0.89)					
Cut Point (OD)	Sens (CI)	Spec (CI)	PPV (CI)	NPV (CI)	Agreement (CI)
≥0.1295*	69% (52;83)	68% (43;87)	82% (65;93)	52% (31;72)	69% (55;80)
≥0.148†	46% (30;63)	95% (74;100)	95% (74;100)	46% (30;63)	62% (48;74)
≥0.18‡	31% (17;48)	95% (74;100)	92% (64;100)	40% (26;56)	52% (38;65)

(2A) compares the definite TB meningitis (n = 39) and unselected non-TB meningitis (n = 54) groups. To evaluate whether other concomitantly used rapid tests could enhance the specificity of the LAM assay the data were also analysed when the non-TB groups were divided into those who had a positive Gram stain or CLAT (2B), or those non-TBM patients who had a negative Gram stain or CLAT (2C). Each of the tables (2A, 2B, 2C) has results specified using the manufacturer's cut-point, optimal cut point using Youdens index and an AUC derived cut point.

*Values expressed as percentages using manufacturers cut point for urine.

† Optimal cut-point as defined by Youden's index [31].

‡ Cut points chosen from the ROC curve to derive greater utility of LAM as a rule-in test.

# AUC  =  Area under the curve.

Sens  =  sensitivity, Spec  =  specificity, PPV  =  positive predictive value, NPV  =  negative predictive value.

When the definite and probable TBM groups were combined and compared to the non TBM, and using the cut-point of ≥0.18 mg/ml, the outcome data were as follows: sensitivity 14% (95% CI:8,23), specificity 94% (95% CI: 85,99), PPV 81% (95% CI: 54;96), NPV 39% (95% CI: 31;48) and agreement 44% (95% CI: 36;52). When these LAM data were combined with the clinical index at a cut point of ≥6, the sensitivity and agreement improved significantly to 38% (95% CI: 28;49, p =  <0.0001) and 58% (95% CI: 50;66, p = 0.01), respectively. The specificity, PPV and NPV was 93% (95% CI: 82;98), 90% (95% CI: 76;97) and 47% (95% CI: 37;57), respectively.

There were 19 patients in the non-TBM group in whom the LAM antigen was positive using the OD value of 0.1295 as the cut-point. Of this group 14 patients had cryptococcal meningitis and 5 patients had viral meningitis. Given that patients were transferred out to referring hospitals upon improvement no comprehensive follow-up information was available for these patients. Twelve patients in the definite TBM group were negative for LAM antigen detection.

### Relationship to CD4 count

When patients were stratified according to CD4 count, there was a greater likelihood of definite TBM when the CD4 count was <100 cells/µl versus ≥100 cells/µl; p = 0.01; Table 3. A comparison of CD4 counts <200 to ≥200 cells/µl also showed significance; p = 0.03. No significant difference was seen when sensitivity was compared in the in the HIV-infected and HIV uninfected groups (p = 0.3; Table 3). However, there were very few patients in the HIV negative group.

**Table 3:** LAM performance outcomes in definite TBM and non-TBM patients when stratified by HIV status and CD4 count.

	All (95% CI)	HIV * (95% CI)		CD4 [All] (95% CI)		
		Negative	Positive	<100 ‡	100–199 ‡	≥200 #
	(n = 93)	(n = 10)	(n = 81)	(n = 39)	(n = 23)	(n = 31)
Sensitivity†	31(17;48)	0% (0;60)	35 (20;53)	50 (27;73)	18 (2;52)	0 (0;37)
Specificity†	94 (85;99)	100 (54;100)	96 (86;99)	95 (74;99)	100 (74;100)	91(72;99)
PPV†	80 (52;96)	N/A	86 (57;98)	91 (59;99)	100 (16;100)	0 (0;84)
NPV†	65 (54;76)	60 (26;88)	67 (55;78)	64 (44;81)	57 (34;78)	72 (53;87)
Agreement	68 (57;77)	60 (26;88)	70 (59;80)	72 (55;85)	61 (39:80)	68 (49;83)

*Comparison between HIV positive and HIV negative patients was not significant; p value was 0.3.

† Expressed as percentages.

‡ Comparison between sensitivity values for CD4 counts <100 with ≥100, the p value was 0.01.

#Comparison between sensitivity values for CD4counts <200 with ≥200, the p value was 0.03.

Note: HIV status was unknown for 1 patient in the definite TBM group and 1 patient in the non TBM group.

There was no distinction when comparing probable TBM and non TBM groups when stratified according to a CD4 count <100 cells/µl versus ≥100 cells/µl; p = 0.4.

### Derivation of a clinical prediction rule and comparison to CSF LAM levels

We derived a clinical index using univariate and multivariate analysis (shown in Table 4). Factors significantly associated with a diagnosis of TBM in the multivariate analysis were CSF to serum glucose ratio, lymphocyte count, CD4 count and a negative CLAT result. The clinical prediction rule was then calculated using the formula based on the coefficients from the multivariate model and examined using various cut-points for the diagnosis of TBM. The cut-point of ≥6 provided the best rule-in value. Table 5 shows outcome data using the clinical prediction rule alone and when the clinical prediction rule was combined with the LAM result: there was a significant improvement in sensitivity (31% to 63%; p =  <0.001) and agreement (68% to 80% (p = 0.01) but specificity remained high at 93%.

**Table 4:** Univariable and multivariable analysis for the prediction of definite TB meningitis.

Characteristic	OR	95%CI	p value	β coefficient	Score
**Univariate analysis**					
Lymphocyte count >200 (cells/µl)	6.5	(2–22)	0.003		
Neutrophil count ≥36 (cells/µl)	5.0	(2–12)	<0.001		
Protein Level ≥2.5 g/l	3.6	(1–10)	0.02		
CSF glucose ≤1 mmol/l	8.4	(3–24)	<0.001		
Ratio of CSF/serum glucose ≤0.2	9.3	(3–28)	<0.001		
CD4 count (<200 cells/µl)	2.9	(1–7)	0.03		
CLAT test (NEG)	8.7	(3–28)	<0.001		
Previous TB (no)	3.1	(1.2–8.0)	0.02		
**Multivariate analysis**					
Ratio of CSF/serum glucose ≤0.2	7.1	(1.8–29)	0.006	2	2
Lymphocyte count >200 (cells/µl)	7.6	(1.5–40)	0.017	2	2
CD4 count (<200cells/µl)	6.8	(1.9–24)	0.003	1.9	2
CLAT test (NEG)	12.9	(3–52)	<0.001	2.6	3

**Table 5:** Comparative performance outcomes of the clinical prediction rule, LAM, and a combination of LAM and the clinical prediction rule for the diagnosis of definite TB meningitis.

Definite TBM (n = 38)* compared to unselected Non TBM (n = 54)						
Cut Point	Sens	Spec	PPV	NPV	Agreement	AUC
	(CI)	(CI)	(CI)	(CI)	(CI)	(CI)
CPR† ≥4	87%	70%	67%	88%	77%	86%
(excluding LAM)	(72;96)	(56;82)	(52;80)	(75;96)	(67;85)	(79;94)
CPR (≥6)	47%‡	98%	95%	73%	77%	0.86
(excluding LAM)	(31;64)	(90;100)	(74;100)	(61;82)	(67;85)	(0.79;0.94)
LAM (OD) ≥0.18	31%#	94%	80%	65%	68%	0.74
	(17;48)	(85;99)	(52;96)	(54;76)	(57;77)	(0.64;0.84)
CPR (≥4) + LAM	89%	65%	64%	90%	75%	77%
	(75;97)	(51;77)	(50;77)	(76;97)	(65;83)	(69;85)
CPR (≥6) + LAM	63%‡, #	93%	86%	78%	80%	80%
	(46–78)	(82;98)	(67;96)	(66;87)	(71;88)	(0.71; 0.88)

*One patient did not have lymphocyte count and was excluded.

† Clinical prediction rule.

‡ p value comparing sensitivity of the clinical prediction rule alone (47%) vs. the clinical prediction rule plus the LAM result (63%) = 0.07.

#p value comparing sensitivity LAM 31% vs. clinical prediction rule combined with LAM (63%) <0.001.

Sens  =  sensitivity, Spec  =  specificity, PPV  =  positive predictive value, NPV  =  negative predictive value.

### Comparison of rapid tests for the diagnosis of TBM

Three tests were applied for a rapid diagnosis. When examining for yield for rapid diagnosis using *only* culture positive patients as the denominator (n = 31 of the 39 definite TBM patients) the yields for smear microscopy, LAM and PCR were, 0 (0%), 9 (29%, at cut-point ≥0.18) and 14 (45%), respectively. Thus, there was a significantly improved yield over smear microscopy when using both LAM (p<0.001) and PCR (p =  <0.001). There was no significant difference between the LAM and PCR yield (p = 0.22).

## Discussion

This study has three major findings. Firstly a newly developed clinical prediction rule, suited to resource-poor high HIV prevalence settings, is a useful rule-in test for the rapid diagnosis of TBM. Secondly, LAM antigen, which has not previously been prospectively evaluated in CSF, is useful as a rapid rule-in test for the diagnosis of TBM in HIV-infected individuals with advanced immunosuppression. Thirdly, combining the prediction rule with LAM antigen detection further increases the rule-in value for TBM.

Given that standardised PCR assays have modest sensitivity [12], are expensive, and not widely available in high TB and HIV burden settings, smear microscopy remains the only diagnostic test that can rapidly and confidently establish a diagnosis of TBM. Thus, although at first glance, the LAM sensitivity of 31% may seem modest the high specificity confers rule-in value enabling a rapid diagnosis in approximately a third more of patients than could have been obtained with microscopy. Furthermore, in HIV-infected persons with a CD4 count of less than 100 cells/µl the sensitivity rose to 50%. Several studies have now confirmed the rule-in utility of the LAM ELISA in HIV co-infected subjects with advanced immunosuppresion when using urine samples [19], [21], [24], [25], [32], [33]. These studies have also confirmed that urine LAM positivity is associated for HIV positivity and advanced immunosuppresssion characterised by low CD4 counts [19], [24], [32]. Similarly, we confirm that in CSF LAM positivity is associated with a positive HIV status and low CD4 count. We hypothesise that HIV-infected patients, particularly those with advanced immunosuppression, have a higher mycobacterial load and hence higher levels on LAM antigen in body fluids [24].

These results are of considerable significance given that a POC lateral flow format of the LAM assay is now at a finalised prototype stage and available for clinical trials [21]. This POC format now requires optimisation using CSF given that the assay was designed for use with urine samples.

A clinical prediction rule using laboratory parameters widely available in resource-poor high HIV prevalence settings had rule-in value similar to that of the LAM assay. An existing clinical prediction rule has been developed by Thwaites and colleagues but was tailored to diagnose bacterial rather than TB meningitis [9]. Similarly, two other studies in resource-poor settings identified a week long history, CSF cell count of <1000 cells/mm^3^, predominant lymphocytosis, and focal deficits as predictive for TBM [34], [35]. These studies sought to distinguish bacterial and TBM, rather than distinguishing between causes of aseptic meningitides. More recently, case definitions for TBM in high burden settings have been proposed [36]. We applied these definitions to our probable TB group. This categorised patients as probable TBM (64%; based on the proposed definition as outlined in [36]) and as possible TBM (35%; based on the proposed definition as outlined in [36]). When our scoring system was combined with the LAM assay the rule-in value significantly improved to ∼65%. Thus, if the POC version of the LAM assay is combined with the prediction rule then this could likely enable the rapid diagnosis of definite TBM in more than two thirds of HIV co-infected patients in an African setting. Preliminary data suggest that the POC LAM assay has equivalent sensitivity to the ELISA version when using urine samples [21]. The assay now requires testing in CSF.

Albeit below our ROC-derived cut-point of 0.18 LAM antigen was nevertheless detected in 19 patients in the non-TBM group (14 cryptococcal meningitis and 5 viral meningitides). Seven patients in this group had culture proven dual infection, i.e. tuberculous and cryptococcal meningitis, which is well documented in HIV-infected patients [37]. In the remaining 12 patients the possibility of concomitant TB infection cannot be excluded with certainty and thus misclassification bias may explain the detection of LAM antigen, at least, in some of these patients. Although comprehensive follow-up was not available, these patients were observed as in-patients for a period of 7 to 10 days for improvement before discharge back to their referring hospitals. We believe that misclassification bias is unlikely to fully explain our observations as the same cohort segregated well when the CSF samples were applied to the antigen-specific ELISPOT test [28]. An alternative explanation might be cross-reactivity with cryptoccocal antigen. Indeed, mannan residues are found in the crytococcal cell wall [38]. However this possible explanation is unlikely as there was no change in specificity when cryptococcal meningitis was excluded from the comparison between definite and non-TBM groups. Thus, the most likely explanation is cross reactivity to another antigen, possibly a host-derived lipid antigen, which is found in high concentrations in the lipid-containing central nervous system tissue. The sensitivity and specificity is different in non-lipid containing biological fluids remote from the site of infection such as urine.

The 12 false negative LAM test results in the definite TBM group are more difficult to explain. Possibilities include, given the paucibacillary disease, a low concentration of LAM antigen in the CSF, which was below the detection limit of the assay, and the sequestration of LAM antigen in immune complexes despite the heating step designed to separate LAM-antibody complexes [39].

Previous studies have documented yields of ∼60% for smear microscopy of the CSF after careful examination for 30 minutes and using high volume processed samples [40]. By contrast, our study similar to others, performed in a programmatic setting [6], [10], [40], [41], [42], [43], [44], [45], [46] detected virtually no positive samples by smear-microscopy. The high workload (300 microscopy slides per day) by a single technician with brief microscopic examination of small volumes of CSF in a ‘real world’ setting prevents reproduction of yields seen in a research setting [40]. Thus, our study highlights the difficulty of making a diagnosis of TBM in a high HIV prevalence setting and the need for new diagnostic tools. Although lymphocytosis predominates in 85% of patients [6], [7], [9], [10], [35], [45], [47], [48], [49], [50] a neutrophil predominance may be seen in the first 10 days [51] (even in HIV co-infected patients [50], [52]), the CSF may be acellular, and the protein levels normal [47]. Thus a variable picture leaves one uncertain when faced with a patient requiring empirical therapy.

The limitations of this study are the small sample size and inadequate follow up of patients to better characterise patients in terms of diagnosis. However, this study is relatively large compared to others evaluating diagnostic tools for TBM. Furthermore, our results are only generalisable to African populations with a high HIV prevalence, and these results remain to be confirmed in different geographical settings. Finally, it should be borne in mind that this commercial assay is optimised for use using urine samples and its use here represents an off-label indication. Further studies are now required using an ELISA and lateral flow assay optimised using CSF samples.

In summary, this study suggests that a standardised LAM antigen detection assay is a useful rapid rule-in test for TBM in HIV-infected individuals with advanced immunosuppression. The test's rule-in value can be enhanced by combining it with a clinical prediction rule developed for patients with suspected TBM from a high TB and HIV prevalence setting. Studies are now required to confirm these findings in other settings, to ascertain the value of combining LAM with alternative rapid tests such as quantitative T cell assays [28], and to clarify the utility of using a lateral flow point-of-care LAM assay optimised for use with CSF.
